# Network Modeling Identifies Patient-specific Pathways in Glioblastoma

**DOI:** 10.1038/srep28668

**Published:** 2016-06-29

**Authors:** Nurcan Tuncbag, Pamela Milani, Jenny L. Pokorny, Hannah Johnson, Terence T. Sio, Simona Dalin, Dennis O. Iyekegbe, Forest M. White, Jann N. Sarkaria, Ernest Fraenkel

**Affiliations:** 1 Department of Biological Engineering, Massachusetts Institute of Technology, Cambridge, Massachusetts, 02139, USA; 2 Department of Radiation Oncology, Mayo Clinic, Rochester, Minnesota 55905, USA; 3 Koch Institute for Integrative Cancer Research, Massachusetts Institute of Technology, Cambridge, Massachusetts 02139 USA

## Abstract

Glioblastoma is the most aggressive type of malignant human brain tumor. Molecular profiling experiments have revealed that these tumors are extremely heterogeneous. This heterogeneity is one of the principal challenges for developing targeted therapies. We hypothesize that despite the diverse molecular profiles, it might still be possible to identify common signaling changes that could be targeted in some or all tumors. Using a network modeling approach, we reconstruct the altered signaling pathways from tumor-specific phosphoproteomic data and known protein-protein interactions. We then develop a network-based strategy for identifying tumor specific proteins and pathways that were predicted by the models but not directly observed in the experiments. Among these hidden targets, we show that the ERK activator kinase1 (MEK1) displays increased phosphorylation in all tumors. By contrast, protein numb homolog (NUMB) is present only in the subset of the tumors that are the most invasive. Additionally, increased S100A4 is associated with only one of the tumors. Overall, our results demonstrate that despite the heterogeneity of the proteomic data, network models can identify common or tumor specific pathway-level changes. These results represent an important proof of principle that can improve the target selection process for tumor specific treatments.

Glioblastoma multiforme (GBM) is the most common and aggressive type of brain cancer in adults, and despite radiotherapy and chemotherapy, median survival is only 12–15 months. Patient tumors are extremely heterogeneous^1^,^2^, and understanding this diversity is likely to be critical for determining potential therapeutic targets and patient-specific treatment strategies^1^. Using comprehensive genome-level characterizations and transcriptional analyses, the Cancer Genome Atlas (TCGA) has classified GBM into subtypes based on gene expression, mutation and DNA copy number data^3^. The vast majority of GBM tumors (88%) show disruptions in tyrosine kinase signaling, most frequently occurring in *EGFR*^4^. In about 50% of GBM cases, *EGFR* is amplified; of these, about half have deletion of exons 2–7, called the *EGFRvIII* mutant^3^,^4^. Proteomic analyses have begun to reveal the complexity of signaling changes in GBM. Johnson *et al*.^5^ performed a high-throughput analysis in xenograft models of GBM generated from patient surgical samples having different EGFR status to quantify the signaling system and to evaluate *EGFRvIII*-mediated signaling. This analysis showed that the signaling behavior and tyrosine phosphorylation levels are heterogeneous across different tumor lines and downstream signaling is not related in any obvious way to EGFR status for the analyzed lines.

The extremely complex molecular changes occurring in GBM require a systems-level analysis to identify potential therapeutic strategies. Several data analysis and network modeling efforts have been used to identify cancer pathways and signaling networks^6^,^7^,^8^,^9^,^10^,^11^,^12^,^13^,^14^,^15^,^16^,^17^,^18^,^19^,^20^,^21^. In an integrated study, gene expression and DNA copy number data were combined with protein-protein and protein-DNA interactions to identify causal genes driving GBM generation *in vitro*^16^. Recently, epigenomic, phosphoproteomic and transcriptome changes induced by *EGFRvIII* have been integrated in a cell line model of GBM, and the reconstructed network successfully revealed several novel targets whose inhibition is effective in blocking GBM growth *in vitro*^22^.

In this work, we develop a strategy to address a critical question for tumor specific therapy: can network-based approaches reveal patient specific pathways and targets? We hypothesized that although the proteomic data obtained by Johnson *et al*.^5^ are heterogeneous, network reconstruction of these data could reveal cancer pathways that are altered in all or a subset of the patients. We used the solution of the prize-collecting Steiner forest (PCSF) problem^23^ to map *in vivo* phosphorylation events from xenograft models of eight tumor lines^5^ onto a network of physical interactions. The PCSF approach uses the significance of the phosphoproteomic events and the reliability of each reported protein-protein interaction to obtain small, high-confidence networks for each tumor. Further, by integrating all human cell surface receptors (receptome) and orienting the signal from receptors to downstream phosphorylation events, we are able to determine the direction of signaling in the networks. The reconstructed disease networks illustrate multi-receptor mediated signaling with shared downstream pathways in GBM tumor lines.

We applied network-based approaches to prioritize targets for experimental tests. Comparison of the disease networks identified a highly confident set of hidden common pathways and proteins. These included the dual specificity mitogen-activated protein kinase kinase 1 (MEK1) and mTOR pathway components. MEK1 is centrally located in the network models of all tumor lines and validation results showed that phosphorylation of MEK1 was increased in all tumors relative to normal human astrocytes. The network approach also predicted that NUMB was present in a subpopulation of the tumor lines. We revealed a significant relation between NUMB presence in the reconstructed networks and clinical data, and showed that NUMB is a marker of invasiveness. In addition, the metastasis mediator S100A4 is found to be a patient-specific target that associated with only one of the tumors. These results demonstrate the utility of network-based modeling for identifying potential targeted therapies in xenograft models.

## Results

### Network Modeling of GBM Phosphoproteomic Data Revealed Altered Signaling Pathways

Phosphoproteomic data measure very different molecular processes from the transcriptional data that have historically been used to classify GBM tumors into four subtypes, namely mesenchymal, proneural, neural and classical subtypes. To understand the relationship between these two types of data, we searched for TCGA tumors that had been assigned to a subtype based on transcriptional data and for which phosphoproteomic data were also available. Then, we clustered the TCGA samples based on the phosphoproteomic profiles and tested whether the assigned subtypes were still in the same cluster or not. The results showed that the phosphoproteomic data do not track with the assigned GBM subtypes in TCGA (Figure S1). This observation suggests that the phosphoproteomic data are highly complementary to the transcriptional data, and may provide insights that are not available from an analysis of the transcriptional data alone.

In the rest of this study, we analyzed a very detailed phosphoproteomic study of eight xenograft models of GBM established by implanting human patient specimens directly into nude mice^5^. These mass-spectrometry-based data quantified 1588 proteins and 225 tyrosine phosphorylation sites on 168 proteins across eight tumor xenografts. The xenograft models have been studied extensively with regard to their responses to different treatments and the status of some biomarkers, such as PTEN, EGFR and p53^24^,^25^,^26^,^27^,^28^,^29^. The original patients were clinically diverse, and the tumors showed a wide range of responses to treatments (Supplementary Text, Figure S2A). The clinical data also include twelve other xenograft models from the same center (Mayo Clinic). The proteomic data have been previously published and analyzed^5^, and the authors noted that the phosphoproteomic signals did not correlate with mutational status (Figure S2B). Additionally, clusters obtained from the phosphoproteomic signals were enriched in very generic biological processes such as phosphorylation and transmembrane receptor protein tyrosine kinase signaling pathway.

Although the phosphoproteomic and clinical data of these GBM xenografts are obviously heterogeneous, and phosphoproteomic profiles do not track with the known GBM subtypes, we asked whether there might nevertheless be some common underlying pathway changes in all or a subset of the tumors that could form the basis for therapies. To search for such pathways, we took the advantage of our previously published network modeling strategy based on the solution of the PCSF problem^23^. This approach searches for high-confidence regions of the global protein-protein interaction network that contain many phosphoproteomic hits. Using this framework, we separately analyzed phosphoproteomic data from xenograft models for each of the eight patients. Naïve methods such as considering first or second-degree neighbors of experimental hits usually give a hairball-like structure (see Figure S3). The PCSF solution yields a better representation of the experimental data by balancing two objectives. On the one hand, it tries to include as many of the proteomic hits as possible; on the other hand, it tries to keep the network small by avoiding using unreliable protein-protein interactions. Specifically, each protein identified as changing in phosphorylation status was assigned a “prize” equal to the absolute value of log-fold change in phosphorylation in each tumor line normalized to the mean of all eight lines. When the algorithm includes a protein in the network, it keeps the prize for that protein, but also has to pay a cost for the protein-protein interaction that links the new protein to the rest of the network. The cost is calculated from the interaction scores representing confidence the confidence that an interaction is real; interactions with higher costs are less reliable. By seeking to maximize the collected prizes and minimize the edge costs, the PCSF approach uncovers the most confident networks linking the experimental data.

We used two variations on the core PCSF approach to ensure that the results would be biologically plausible signaling networks. First, we required that each subnetwork identified in the PCSF solution starts at a cell surface receptor. Second, we accounted for the significant cross-talk between different signaling pathways in mammalian signaling by systematically searching for alternative pathways consistent with the data. To find cross-talk among pathways, we performed *in silico* knock-out experiments in which we removed the optimal cell surface receptor and searched for an alternative network (detailed in Methods). This approach identified other cell surface receptors sharing downstream pathways with the optimal receptors (see Figure S3). At the end of all perturbations, the individual solutions were merged to generate the final network. Using this approach, we reconstructed eight final disease networks representing individual tumor lines (labeled as GBM6, GBM8, GBM10, GBM12, GBM15, GBM26, GBM39, GBM59).

We also developed target selection strategies for prioritizing nodes in the networks. First, we defined a “specificity index” to identify proteins in the resulting networks that are specific to the disease pathways. For each tumor line, we generated 100 random inputs, determined the optimal PCSF solution, and computed the frequency that a protein appeared in these control calculations. The approach illustrated in Figure S4 (and see *Methods*), labels each node in the final networks with the frequency at which it is detected in the control calculations. Proteins with a high probability of appearing in random networks included hubs such as p53, EGFR, GRB2, CBL, INSR, AR, PTPN1, etc., some of which undoubtedly are part of the altered pathways, but may have off-pathway effects as well. This “specificity index” can be used like a p-value to focus experimental efforts on the proteins most likely to be highly relevant to the disease.

Our second strategy for selecting targets evaluated information flow in the network, using the concept of network damage. We systematically determined how loss of an individual node would alter signaling by calculating the path damage ratio, which is based on the number of paths from the cell surface receptors to terminal proteins that were disrupted (see Figure S5a and Methods). We also applied other schemes for ranking targets using multiple centrality measures such as degree centrality, betweenness centrality, eigenvector centrality, pagerank and closeness centrality (see Figure S5b and Methods). The final selection of targets was determined by filtering for nodes by the specificity index, and the centrality features. The list of top ranking proteins for each patient (calculated by the union of the path damage ratio and centrality rankings) was organized into a matrix representation and presented in Figure S6. Each protein is colored according to the specificity index. The most common and central proteins are also listed in Table S1.

### Example of a disease network

**Figure 1:**
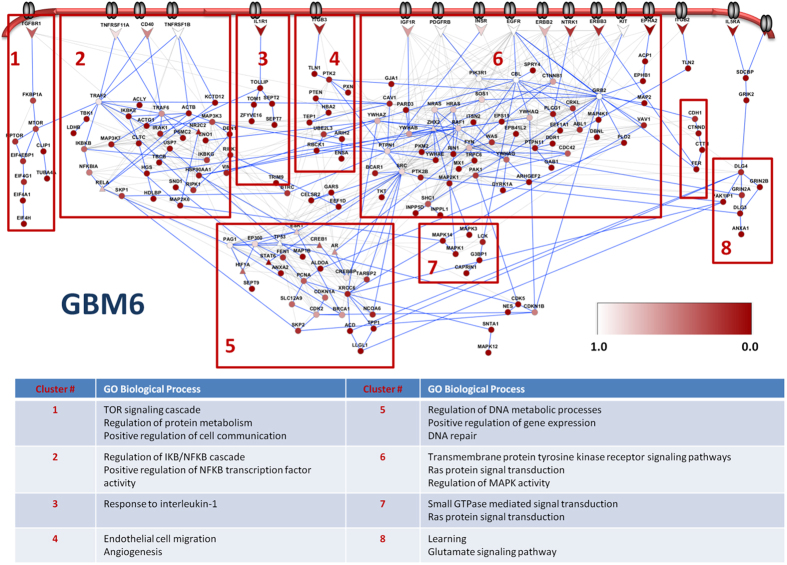
The disease network of GBM6. Nodes are clustered with the edge-betweenness clustering algorithm. Each cluster is enriched in several biological processes, which are tabulated in the lower panel of the figure. Nodes are colored according to the probability they are present in the random networks. Arrow shaped nodes are cell surface receptors. For an interactive visualization of GBM6 and other tumor lines’ networks, browse http://fraenkel.mit.edu/steinernet/xeno_output/results/main.html.

**Figure 2:**
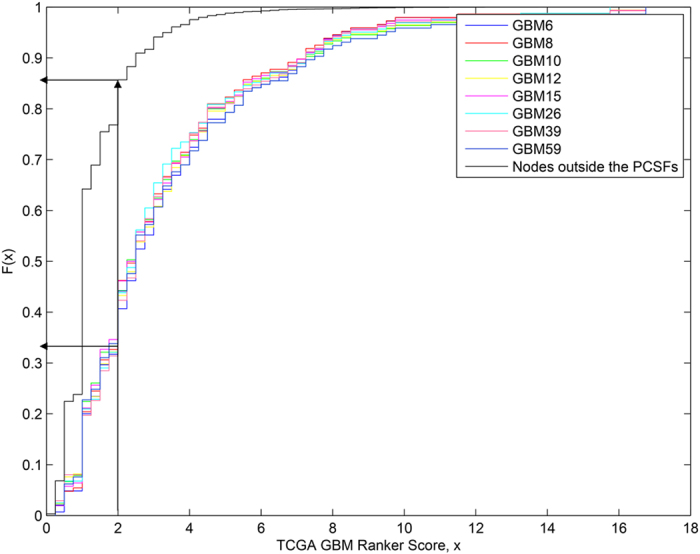
Assessing the relevance of the PCSF solutions to GBM. The cumulative distribution function is plotted for the GBM GeneRanker scores for each tumor line and for the nodes outside the PCSFs. (Higher scores indicate greater evidence of association with GBM.) As the arrows point out, the probability of having a score less than 2.0 for proteins within the disease network is 0.35 on average while this probability is 0.85 for the nodes outside the PCSFs.

The disease network of GBM6 that we obtained by this approach is illustrated in Fig. 1, where we have indicated the specificity of the proteins to the disease and identified sub-networks using edge-betweenness clustering (see Methods). The same network with a different coloring scheme is shown in Figure S7 to distinguish phosphoproteomic hits (terminal nodes) and intermediate proteins, called Steiner nodes. The algorithm connects the terminal proteins either directly or through Steiner nodes, which are derived from the interactome and are relevant to the biological response in question, but were not detected in the high-throughput experiments. The sub-networks contain both known GBM-related pathways and novel aspects of the disease. Examples of known GBM-related pathways recovered are the canonical signaling Ras/Raf/MAPK cascade, and the PI3K signaling pathways as well as DNA repair and cell cycle processes. Out of around 500 cell-surface receptors, only ~3% of them were included in the final network. Among those that were included, EGFR, PDGFRB, and ERBB2 are well-known receptors that function in a cooperative manner in GBM. The TNF family of receptors mediates several signal transduction mechanisms that result in cell apoptosis or gene up-regulation via the MAPK and NFkB pathways. In addition, the Src family kinases (SFKs) are located immediately downstream of the receptor tyrosine kinases (RTKs) in our network, and are well known to be activated and highly expressed in GBM tumors^30^,^31^. We also systematically assessed the relevance of the recovered networks to GBM using the TCGA Gene Ranker (http://cbio.mskcc.org/tcga-generanker/), which combines data on gene expression, mutation, pathway analysis and literature curation. The scores of the nodes in our disease networks are higher than the rest of the interactome. The probability of selecting a high-scoring protein in the modeled networks is 0.66, while the probability is 0.15 for the rest of the interactome (Fig. 2). Overall, these results show that the recovered networks capture many known aspects of GBM.

### Comparison of the disease networks across GBM samples revealed hidden common pathways and clinically relevant proteins

**Figure 3:**
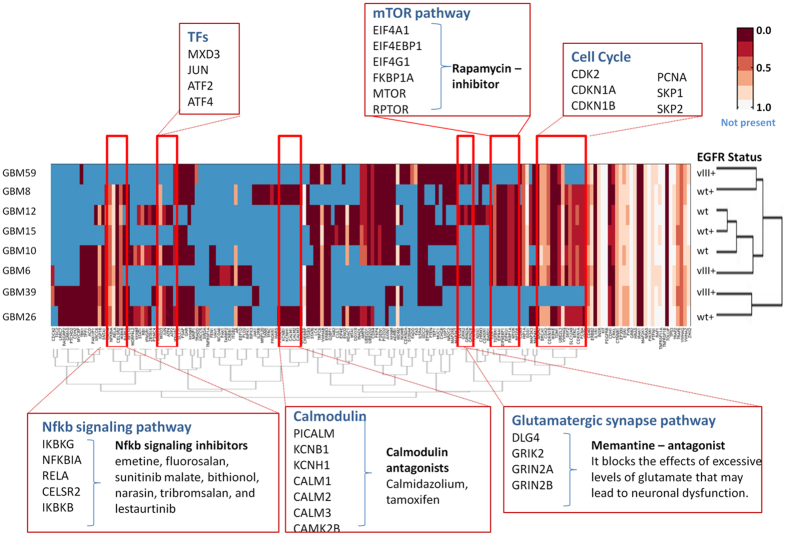
Hidden nodes identified by network analysis for each xenograft line. Each reconstructed network was converted into a binary matrix where columns and rows represent proteins and tumor lines. If a protein is not present in the network of a tumor line the corresponding entry is colored blue. Otherwise, the corresponding entry is colored according to the specificity index. Saturated bars represent more confident regions that are more specific to the GBM data. Less saturated bars represent very generic pathways and essential proteins.

We next evaluated whether the network models could identify novel disease-related proteins. Specifically, we selected sets of proteins that were implicated by our algorithm as affected in particular tumors, but that were not directly observed in the experiments. These hidden components of the cellular response are known as “Steiner nodes.” We used a heat-map to evaluate the association of Steiner nodes with the eight tumor lines, where columns represent Steiner nodes, rows represent tumor lines and entries indicate whether or not the protein was present and its specificity index in the PCSF solution (Fig. 3). Clustering this matrix revealed several pathways and proteins common across multiple tumor lines while some others are only present in one tumor line. After filtering using the specificity index described above, we were also able to identify Steiner nodes that are found in all the GBM data we analyzed, but still rare in control calculations. These included components of the NFkB signaling pathway, the mTOR pathway, the glutamate signaling pathway, calmodulins, and multiple transcription factors. We note that these pathways and groups were not present in the experimental data; they were only revealed by our network modeling.

The clustering based on common pathways was independent of tumor *EGFR* status. We were able to observe several interesting results based on these commonalities in the network models of GBM tumor lines. For example, several transcription factors were shown to be grouped together including ATF2, ATF4, JUN and MXD3. Among them, JUN functions in cell proliferation and cell death, and is aberrantly expressed in GBM^32^. Also, ATF family transcription factors have crucial roles in tumorigenesis in different types of cancers^33^. Another example of commonalities across the tumor lines is the glutamatergic synapse pathway, components of which were revealed as common in three out of eight patients. Glutamate secretion stimulates tumor growth, proliferation, and survival through activation of the mitogen activated protein kinase and the PI3K/AKT pathways^34^. Glutamate is a neurotransmitter that modulates synaptic transmission via the glutamate receptor GRIN2A and GRIN2B. Dysfunction of glutamate signaling was shown to be frequent in gliomas^35^. We found that components of the mTOR signaling pathway were present in five (GBMs 6, 8, 10, 12 and 15) out of eight tumor lines. Previous studies have shown that the RTK/PI3K/AKT/mTOR molecular cascade is abnormally activated in GBM and functions in the control of cell growth and proliferation. We hypothesize that GBM lines with mTOR pathway components in their network models should be more sensitive to direct mTOR inhibition than those lacking mTOR in their pathways. Overall, the success of the PCSF approach in revealing the known biology of GBM, as well as commonalities across multiple xenograft models, gave us confidence that these networks and collected clinical data of tumors could be used to identify important targets and validate their effect in GBM.

### MEK1 was revealed and experimentally validated as altered in all the tumor lines

**Figure 4:**
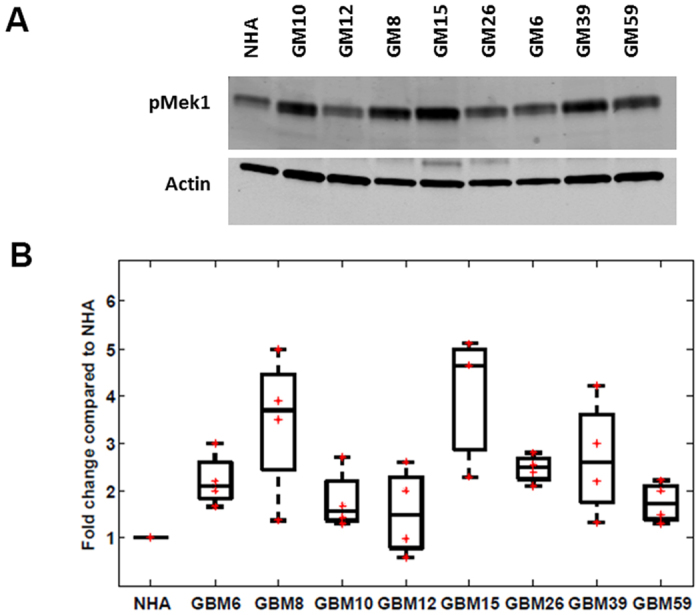
(**A**) Phosphorylated MEK1 Western blotting across NHA and eight human GBM xenograft lines. For all blots, the upper panel shows pMEK1 at Serine 298 and the lower panel indicates the actin control blot. (**B**) Boxplot of phosphorylation ratios of pMEK1 calculated from Western blotting across eight GBM xenograft lines (four biological replicates).

Using the target selection criteria defined above, we selected MEK1 (*MAP2K1*) as one of the proteins to be validated. MEK1 is a dual-specificity threonine/tyrosine kinase that functions in the activation of the Ras/Raf/MEK/ERK pathway regulating cell growth and is upregulated in a variety of tumor cell types. MEK1 also plays an important role in downregulating expression of O6-methylguanine DNA methyltransferase (MGMT), which is a well-known factor influencing chemotherapy resistance in GBM^36^. MEK1 is present in the network models of all tumor lines, scores well on the specificity index, and is located in the central parts of the networks. We carried out Western blotting experiments for the phosphorylation status of MEK1 in all tumor lines and in normal human astrocytes (NHA) using four bio-replicates for each tumor line. These experiments demonstrated that MEK1 phosphorylation was significantly increased across all 8 GBM tumor lines compared to normal human astrocytes (NHA), except in GBM12 where the results varied across biological replicates (see Fig. 4). We also examined total MEK1 levels to assess whether the observed changes in phosphorylation were affected by alterations in protein abundance. Western blotting experiments showed that only GBM15 had a significant increase in the total amount of MEK1 (Figure S8). These results revealed that although MEK1 was not included in the initial experimental data, our network modeling approach successfully recovered MEK1 as an activated protein common to all tumor lines. Combined with the fact that MEK1 is essential for regulation of cell growth and downregulates MGMT^36^, our network results suggest that single agent MEK1 inhibitor therapy or concurrent MEK1 inhibition and chemotherapy may be good strategies to be tested in GBM tumor lines. Our results show that network modeling approach can nicely complement the missing targets in the phosphoproteomic data as illustrated with the phosphorylation status of MEK1, which was phosphorylated on a serine, and thus could not be detected in the phosphotryosine proteomic study.

### Network modeling also identifies patient specific pathways

**Figure 5:**
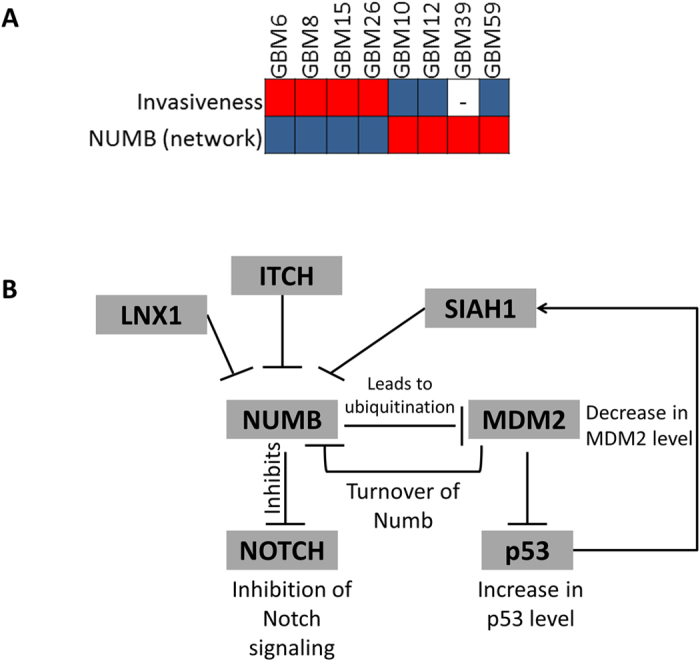
(**A**) NUMB protein as a tumor invasiveness marker. Entries with highly invasive tumors are colored red. Minimally or non-invasive tumor entries are colored blue. Entries with tumors containing Numb in the modeled network are colored red. (**B**) Known NUMB activity leads to ubiquitination of MDM2 and in turn increases p53 level. NUMB activity also inhibits Notch signaling. The first degree neighbors of NUMB in the interactome have other ubiquitin ligases including SIAH1, LNX1 and ITCH.

We also sought to determine whether our approach could identify patient-specific therapies. We searched among the Steiner nodes that satisfy our target selection criteria for hits and that were present in some but not all of the tumors. We next asked whether the presence of nodes in the network might serve as biomarkers of clinical features that we had assembled for this tumor population (Figure S2A). We found that there is a strong correlation between the presence of NUMB in disease networks and the degree of invasiveness of the corresponding tumor determined by growth in other regions of the brain (28). NUMB was a Steiner node in four out of eight final networks, and scored well on the specificity index. Four tumors (GBM6, GBM8, GBM15 and GBM26) without NUMB in their disease networks were classified as highly or moderately invasive (Fig. 5A). NUMB was present in the network of GBM39, but there was no information about the invasiveness of this tumor line. The remaining three tumor lines having NUMB in their disease networks were minimally or non-invasive. To evaluate the reliability of the association between NUMB and invasiveness, we evaluated whether any Steiner node in a random network could predict invasiveness. Specifically, we tested all combinations of 100 random networks for eight tumor lines, generating 8 × 10^16^ random network combinations. As a result, we found that probability for a Steiner node in the random networks to correlate with the tumor invasiveness phenotype is 1.61 × 10^−14^. These results suggest that the presence of the protein NUMB in the networks is a significant tumor invasiveness marker in xenograft models of human GBM tumor lines.

NUMB is known to function in developmental decisions in two ways: by ubiquitinating MDM2, which results in an increase in p53 levels, and by inhibiting the Notch signaling pathway^37^ (Fig. 5B). We searched for other proteins that interact with NUMB in the initial interactome, and found that NUMB is connected to several other ubiquitin ligases including ITCH, LNX1 and SIAH1. These ubiquitin ligases were previously shown to tightly control the level of NUMB^38^,^39^,^40^. We then checked the relation between genetic alterations of these ubiquitin ligases and NUMB in TCGA using the analysis in cBioPortal^41^. We found that genetic alteration (including deletion, amplification, up/down regulation) in NUMB tend to co-occur with genetic changes in ITCH and SIAH1 (p-values are < 0.001 and 0.002, respectively). Because increased NUMB was correlated with reduced tumor invasiveness, it can be considered as a prognostic target not as a therapeutic target. However, the ubiquitin ligases controlling NUMB expression might be considered as targets for therapeutic inhibition.

### The S100 calcium-binding protein A4 (S100A4) is a tumor-specific target

S100A4 is a metastasis mediator and its over-expression is observed in several types of cancers^42^. Previously, knockdown experiments have shown that inhibition of S100A4 expression suppresses the metastatic capacity of S100A4-expressing tumor cells in animal models. In another study, S100A4 knock down led to upregulation of metastasis-related genes Calveolin-1 (CAV1), MDM2 and E-cadherin (CDH1) in prostate cancer^43^. S100A4 is regulated by many upstream signaling molecules including TGF-β and ERKs. S100A4 has possible effects in cell motility by binding to myosin, in cell adhesion by regulating the expression of CDH1 and in cell proliferation by binding to TP53^44^.

**Figure 6:**
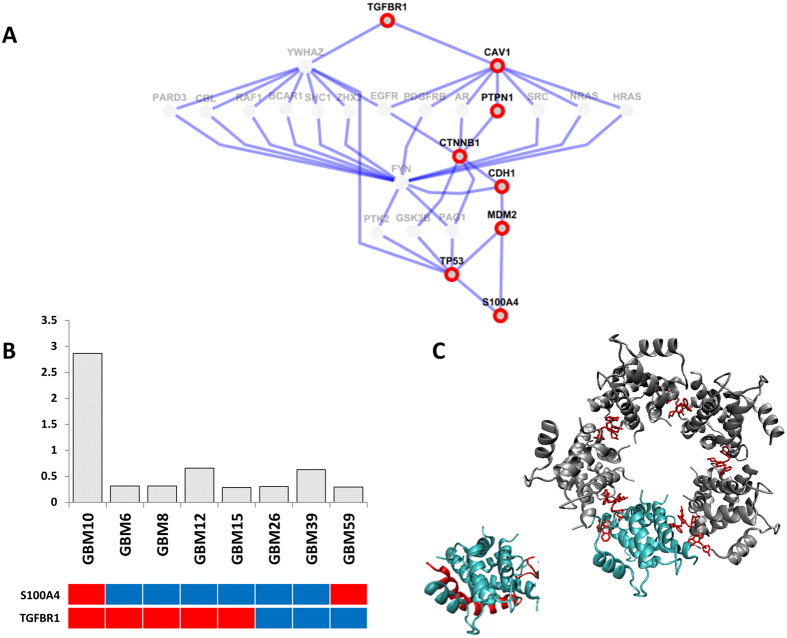
(**A**) The merged network of all simple paths from TGFBR1 to S100A4 in GBM10. Nodes with red border represent the paths connecting many EMT markers including TGFBR1, CAV1, CDH1 and S100A4. (**B**) The upper panel shows the relative S100A4 protein abundances across GBM tumor lines from ref. 5 and the lower panel shows the presence of S100A4 and TGFBR1 in the networks for these tumor lines (red = present, blue = absent). (**C**) The structures of S100A4/Myosin (colored cyan and red, respectively, PDB: 2lnk), S100A4/TFP complexes (S100A4 molecule is colored cyan and other S100A4 molecules in the oligomer state are colored gray, PDB: 3ko0). TFP is in line representation and colored red).

In our network models, S100A4 scored well on the specificity index, suggesting that S100A4 is a high-confidence GBM target. S100A4 was present in the networks of two tumor lines (GBM10 and GBM59)). Finding such a profile across eight tumor lines in randomly generated networks is rare (p-value = 0.0073). We then compared the network results to global proteomic data obtained from the same tumor lines. These data showed that S100A4 was highly over-expressed only in GBM10 (Fig. 6B). In this case, the network results were only partially consistent with the experimental data, but still served to eliminate six out of the seven lines where S100A4 was irrelevant.

We then sought to understand whether, in hind-sight, we could determine network differences between GBM10 and GBM59. Indeed, the network of GBM10 was enriched for epithelial mesenchymal transition (EMT) markers not present in GBM59. In particular TGFBR1, which co-occurs with S100A4 in TCGA data, was only present in GBM10 but not in GBM59. Then, we searched for all simple paths (defined as a path with no repeated nodes) from TGFBR1 to S100A4. Among them, there were some paths connecting many EMT markers starting with TGFBR1 including CDH1, CAV1, and beta-catenin (CTNNB1) and ending with S100A4 in GBM10 (Fig. 6A). Such simple paths were not present in the network of GBM59. These results suggest the value in exploring the full network context of a potential target.

## Discussion

While targeted therapy has dramatically improved outcomes for many cancers^45^, this approach has had limited impact on GBM. Expression profiling and phosphoproteomics have revealed that GBM tumors are extremely heterogeneous, defying any simple classification. In addition, we find that tumors sharing a common transcriptional subtype can have very different proteomic states in the TCGA data, indicating the complexity of these tumors. Nevertheless, it remains possible that there are some commonalities among tumors at the pathway level and some tumor specific targets. We developed a network approach to search for such pathways and targets *de novo*, by identifying regions of the interactome enriched in phosphoproteomic events. By solving the prize-collecting Steiner forest (PCSF) problem for each of eight xenograft models of GBM, we were able to reconstruct tumor-specific models of signaling pathways. We also developed a methodology for choosing targets in these pathways that were likely to be found in GBM networks but rare in control calculations and critical to signal transduction.

Our reconstructed networks revealed several common proteins and pathways across multiple tumor lines. NFkB signaling, mTOR signaling, glutamate signaling and multiple transcription factors are among these common entities. We noted that none of these common proteins/pathways were present in the experimental data; they were only revealed by our algorithm. We conducted experimental tests for a hypothesis based on our reconstructed networks. We examined the role of the dual-specificity threonine/tyrosine kinase MEK1 in these tumors. MEK1 appeared in the networks of all eight tumors, but was extremely rare in random networks. Using Western blotting, we confirmed that phosphorylation of MEK1 was increased in seven of the eight tumor lines compared to normal human astrocytes, with the eighth tumor line (GBM12) displaying a high degree of variability across the four biological replicates. MEK1 plays a critical role in signaling within the Ras/Raf/MAPK cascade and has several important functions in regulating other proteins including MGMT, which is an important modulator of TMZ response^36^. Finally, we assessed whether the network models could predict targets specific to a subset of tumor lines. We revealed that the network approach could identify a biomarker of a clinical feature by showing that the presence of NUMB relates to tumor invasiveness.

Our network approach also identified a highly ranked target, S100A4, that turned out to be specific to a single tumor line. The structure of S100A4/Myosin complex has been recently released. A psychotropic drug, trifluoperazine (TFP), is a member of phenothiazines which binds to S100A4 and induces S100A4 oligomerization. The metastatic capacity of S100A4 is associated with its binding to myosin; and S100A4 in the oligomeric state cannot interact with myosin. In Fig. 6C, structures of S100A4/Myosin and S100A4/TFP complexes are illustrated. In general, TFP is thought to have a synergistic effect with RTK inhibitors such as imatinib, as has been demonstrated in U87 human glioblastoma cell line^46^. These data suggest that a network approach could be used to prioritize patients for a clinical trial with this agent, alone or in combination. We note that for this purpose, the network approaches could likely be improved by incorporating a broader analysis of the S100A4/EMT pathway, rather than focusing on a single node.

In conclusion, our network modeling approach identified several pathways suggesting clinical targets that were not detected in the initial experimental data. Our results demonstrate that the apparent diversity of GBM tumors is not necessarily a barrier to developing targeted therapies for specific subsets of tumors. These experiments should be considered a proof of principle; however, larger scale studies will, of course, be needed to refine the approaches we have described.

## Methods

### GBM Xenografts and Experimental Methods

The animal studies we performed were reviewed and approved by the Mayo Institutional Animal Care and Use Committee and were in compliance with NIH guidelines. We used the same xenografts analyzed in the work of Johnson *et al*.^5^ where human GBM xenografts were established with the injection of 100–200 μL of tumor tissue mixed 1:1 with Reduced Matrigel ectopically into the flanks of nude mice. Each of the eight xenograft lines (GBM6, GBM8, GBM10, GBM12, GBM15, GBM26, GBM39 and GBM59) were derived from primary tumors from different patients undergoing surgical treatment and serially passaged in mice^47^ prior to this study. Four tumor xenografts were generated for each GBM xenograft line. Tumor xenografts were resected from mice and immediately flash-frozen and stored in liquid nitrogen prior to tissue homogenization for Western blotting.

#### Western Blotting

Normal human astrocytes pellet and GBM xenograft lines were lysed and homogenized in lysis buffer (50 mM Tris-HCl, pH 7.4, 150 mM NaCl, 1% NP-40, 0.5% Sodium deoxycholate, 1 mM EGTA) containing protease and phosphatase inhibitors (Thermo Scientific). Total protein content was determined with Bradford assay (Bio-Rad). Extracted proteins were boiled in Laemmli sample buffer for 5 min, separated on 4–20% SDS-PAGE gel (Bio-Rad) and transferred to a nitrocellulose membrane using a liquid transfer apparatus (Bio-Rad). After transfer, the membranes were rinsed and blocked for 1 h at room temperature with Odyssey Blocking Buffer (Li-Cor Biosciences). The membranes were incubated overnight at 4 °C with primary antibodies in Odyssey Blocking Buffer with 0.1% Tween-20: (a) rabbit monoclonal anti-MEK1 (phospho S298) (ab96379, Abcam, dilution 1:1000); (b) rabbit monoclonal anti-MEK1 (ab32576, Abcam, 1:1000); (c) mouse monoclonal anti-actin (MAB1501, Millipore, dilution 1:10000). The membranes were then washed four times for five minutes with TBS-T buffer (10 mM Tris-HCl, 100 mM NaCl, 0.1% Tween-20, pH 7.5) and incubated for 1 h at room temperature with secondary antibodies (dilution 1:10000) in a 1:1 PBS, Odyssey Blocking Buffer solution with 0.5% Tween-20 and 0.01% SDS. The membranes were washed four times for five minutes with TBS-T buffer and then scanned using an Odyssey IR scanner and the Image Studio™ Lite imaging software (Li-Cor Biosciences). Protein expression was measured via integrated intensity readings in regions defined around protein bands and normalized to a control band (actin). Differential expression was calculated as the fold change in normalized protein expression and statistical significance was determined both with a t-test and the Kolmogorov-Smirnov test.

### Configuration of the system for network modeling and optimization

Human protein-protein interactions were retrieved from iRefIndex version 8^48^. Each interaction in this interactome is scored by the MI-score scoring scheme, which considers publications about that interaction, the experimental method for which the interaction was detected, and the type of the interaction to calculate the score. We used a threshold of 0.5 to eliminate low probability interactions in the preprocessing. Since the confidence score s_e_ is log likelihood-based, the cost of edges were calculated as c_e_ = 1−s_e_, so that minimization on the sum of edge costs can be interpreted as maximizing the product of likelihood. We used all identified cell surface receptors in our network modeling design to add directionality implicitly. For this purpose, cell surface receptors were collected from the Human Plasma Membrane Receptome Database^49^.

#### Prize Collecting Steiner Forest Problem

For a given, directed or undirected network *G*(*V, E, c*(*e*)*, p*(*v*)) with node set *V* and edge set *E*, *p*(*v*) ≥ *0* assigns a prize to each node *v* *∈* *V* and *c*(*e*) > *0* assigns a cost to each edge e ∈ E. The aim is to find a forest *F(V*_*F*_*,E*_*F*_) that minimizes the objective function:


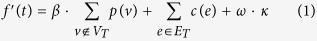


In equation (1), κ is the number of trees in the forest and ω is tuning parameter. A practical way of minimizing *f* ′ consists in casting the PCSF into a PCST (see *SI text*) on a slightly modified graph. The idea is to introduce an extra root node *v*_*0*_ into the network connected to each node *r ∈ R* by an edge (*r*, *v*_*0*_) with cost ω where *R* ⊂ *.V*. In our problem, *R* represents the set of cell-surface receptors. This problem has been solved with the message passing algorithm, msgsteiner tool^7^ on the resulting graph *H*(*V*∪{*v*_*0*_,}*, E* ∪ *.Vx*{*v*_*0*_}) and the solution has been called *T.* We define the forest *F* as *T* with all edges pointing to the root removed. It is straightforward to see that the tree *T* is minimal for *f* if and only if the forest *F* is minimal for *f* ′. The objective function (Eq. 1) is composed of three parts. The first term penalizes networks for excluding experimentally determined proteins; the second term penalizes networks for using unreliable edges; and the third term controls the number of trees in the forest. Thus, minimizing the first two terms balances the desire to include as many experimentally determined proteins as possible with the desire to avoid low-confidence edges. In the objective function (Eq. 1), β is a scaling factor that controls the size of the final network, with larger β value favoring larger networks. The ω parameter is the cost of starting a new tree in the solution, and thus a smaller value of ω results in more trees in the final PCSF. One additional parameter, which is not included in Eq. 1, is the depth (D) of the tree. D controls the maximum number of edges from the extra root node v_0_ to terminal nodes where v_0_ represents an external stimulus that potentially activates multiple receptors. Here, D is set to 10, ω is 0.001 and β is 2.0. Prize of each node in the terminal set is the absolute value of log fold change in phosphorylation in each tumor line normalized to the mean of all 8 channels. If a protein contains more than one quantified phosphorylation site, we selected the weight as the maximum value of the fold changes of these sites.

The resulting network depends on the parameters β and ω. The parameter ω influences the number of subtrees, κ, in the forest, since ω is the cost of each edge that connects to the artificial root. As a result, a very small value of ω results in many sub-trees in the forest, while a very large value of ω results in a single tree rooted by one receptor. The parameter β influences the number of terminals included in the final network, as it controls the trade-off between prizes and edge costs. The final result is an empty solution when β is set to zero, while a very large value of β results in a network that is either empty or constructed with very low probability edges. We have previously described a rigorous method for tuning β and ω^23^ that gives preference to networks in which the parameters that maximize fraction of terminal nodes included in the network, minimize the number of trees and ensure that the trees in the forest are all of similar size.

### Systematic *in silico* Knock-out of the Cell Surface Receptors to Generate a Robust Network

In this work, the term ‘*in silico* knock-out’ means removing the target protein and all its interactions from the whole interactome. During the reconstruction of network models, receptors were knocked out one by one in the first iteration, and in the second iteration double receptor knock-outs were performed. In mammalian signaling systems, there is a high degree of cross-talk between signaling pathways. Because the algorithm searches for the least expensive set of edges to connect the nodes, it will never include redundant paths. As a result, each downstream node will only connect to one receptor. To overcome this situation and to find the cross-talk across different subnetworks, we applied the *in silico* knock-out experiment. Here, a selected receptor and all its interactions were removed from the interactome and the PCSF algorithm was applied to the remaining network. In this way, receptors having shared downstream pathways were distinguishable. This stepwise approach is composed of four stages. The first step is reconstructing the original network. Then, each receptor in the cell surface receptor dataset is knocked-out one by one. Among this set of solutions, we collect all the cell surface receptors and perform a double knockout experiment in the third step. The most striking example to better explain this procedure is the interplay between EGFR and PDGFRB receptors. When we knock-out EGFR the new root for the same tree appears to be PDGFRB. When merge each solution network – the wild type and the knock-out networks- we are able to find shared edges or nodes between different subtrees. In addition, if we consider each KO case as a perturbation in the initial interactome we are also able to include sub-optimal solutions in the final network. Finally, we merge different networks by taking the union of the set of nodes and set of edges in each solution. At the end of this procedure, the network includes multiple solutions and multiple receptors having shared downstream pathways.

### Randomization Tests

We prepared 100 different random terminal sets for each tumor line where each random terminal set contains the same number of proteins with the original terminal set. Node penalties were kept the same and 100 random networks were reconstructed using the original interactome with the original edge weights for each tumor line. In the application of PCSF algorithm, the same parameter set and iterative procedure from the original run were used. To include the sub-optimal solutions we perturbed the initial interactome with in-silico knockout experiments as in the original network modeling step. The probability for a node to randomly appear in any type of network is defined as the frequency of that node in the random networks. For a given original network solution *F(V*_*f*_* , E*_*f*_) where *v*_*f*_ *∈* *V*_*f*_, the probability of *v*_*f*_ to be random, *prob(v*_*f*_), is *n/N where n* is the number of random networks in which *v*_*f*_ is present and N = 100 for the random networks that were reconstructed. We defined a “specificity index” to identify proteins in the resulting networks that are specific to the disease pathways. To calculate the specificity index, we used the probability for a node to randomly appear in any type of network, *prob(v*_*f*_) where specificity index is equal to 1- *prob(v*_*f*_). The detail of this procedure is in Figure S4.

### Network centrality and path damage

We used five different centrality measures (degree centrality, betweenness centrality, eigenvector centrality, pagerank and closeness centrality) and two path damage ratios to rank the nodes in the networks. We utilized the networkx library to calculate the network centralities. As shown in Figure S5b, different centrality metrics give different results about whether a node is central in the network or not. From each list, we selected the top 50 proteins and merged them together.

To calculate the path damage ratios, we sampled all the simple paths from receptors downstream to the phosphorylated/dephosphorylated proteins. We calculated how many paths were damaged when intermediate nodes were removed one by one. To calculate the path damage ratio, the number of damaged paths was normalized with the total number of paths. In this procedure, we used two different approaches. In the first approach, predicted directions of the edges were mapped from the Ben-Shlomo *et al*.’s human interactome^49^ to our network models and path damage ratio was calculated accordingly. Separately, we also used undirected edges and sampled shortest paths from receptors to terminal proteins. At this stage, one edge at each iteration was removed from the shortest path to find the alternative paths and calculated the path damage ratio accordingly.

Figure S5a illustrates a simple example of the path damage concept where the removal of “node A” damages 65% of all paths meaning that “node A” has a very significant effect on the network and behaves like a bottleneck. We calculated the damage ratio for all Steiner nodes in the disease networks and plotted damage ratios versus randomness probabilities for each patient. The intersection of protein sets with randomness probability less than 0.5 and top 50 proteins having highest path damage ratio were added to the targets list.

All seven measures (5 centralities and 2 path damage measures) were considered to find the final target set. If a protein is listed in at least one of the features, it was added to the set.

### Clustering & Enrichment Analysis

We used the edge-betweenness clustering approach described in^12^ to cluster nodes in each network. We first restored all interactions among the nodes in the final network from the original interactome. We then applied edge-betweenness clustering to this augmented network. For the GO enrichment analysis, we used the BINGO plug-in from Cytoscape. All networks generated in this work have been uploaded to http://fraenkel.mit.edu/steinernet/xeno_output/results/main.html. We used the Cytoscape Web plug-in for online visualization. Different node coloring schemes, augmented networks and statistics are available on the website.

## Additional Information

**How to cite this article**: Tuncbag, N. *et al*. Network Modeling Identifies Patient-specific Pathways in Glioblastoma. *Sci. Rep.* **6**, 28668; doi: 10.1038/srep28668 (2016).

## Supplementary Material

Supplementary Information
