# Supplementary material for: Network Modeling Identifies Patient-specific Pathways in Glioblastoma
Source: Sci Rep. 2016 Jun 29;6:28668. doi: 10.1038/srep28668 (PMC4926112; doi:10.1038/srep28668)
Supplement: Supplementary Information [file srep28668-s1.pdf]

Network Modeling Identifies Patient-specific Pathways in Glioblastoma

Nurcan Tuncbag[1], Pamela Milani[1], Jenny L. Pokorny[2], Hannah Johnson[1,3], Terence T Sio[2], Simona Dalin[1], Dennis O Iyekegbe[2], Forest M. White[1,3], Jann N. Sarkaria[2], and Ernest Fraenkel[1,*]

[1] Department of Biological Engineering, Massachusetts Institute of Technology, Cambridge, Massachusetts, 02139, USA
[2] Department of Radiation Oncology, Mayo Clinic, Rochester, Minnesota 55905, USA
[3] Koch Institute for Integrative Cancer Research, Massachusetts Institute of Technology, Cambridge, Massachusetts 02139 USA
*Correspondence should be addressed to (fraenkel-admin@mit.edu)

Supplementary Information

SI Text

Collection of data from published studies for GBM xenograft models from the same center that made the models.

Temozolomide, one of the chemical agents used in GBM therapy, methylates the DNA at O-6 position of guanine which damages DNA and eventually induces tumor cell death. Resistance to the temozolomide therapy has been shown to be modulated by the DNA repair protein O-6-methylguanine-DNA methyltransferase (MGMT) which removes the methyl groups that TMZ added; consequently, expression of MGMT induces resistance to TMZ therapy. We included TMZ response data based on TMZ therapy of 66 mg/kg daily for 5 days. At the end of the TMZ therapy, if the survival ratio is either larger than two-folds or the survival ratio p-value is less than 10^{-4}, that tumor line is labeled TMZ sensitive in our data matrix. Also, expression of MGMT and the status of two other biomarkers (*TP53* mutation and PTEN mutation/deletion) were added to the data for 20 tumor lines (*1*). The response to a mTOR pathway inhibitor, RAD001, has been tested for 17 tumor lines. GBM10 and GMB22 were found to be sensitive to RAD001 (*2*). Another biomarker, MARCKS protein, has been found to be regulator of growth and radiation sensitivity. MARCKS expression is available for only eight tumor lines (*3*). Clinical data such as, response to the radiotherapy treatment, patient age and gender have been also included in our data set for 15 tumor lines (*4*). Treatment with Erlotinib, an EGFR inhibitor, in 12 tumor lines showed that Erlotinib is only effective for GBM10 (*5*). The relationship

between invasiveness of the tumor and the level of E-cad expression has been analyzed extensively for 15 tumor lines previously, which is also included in our data matrix (*6*).

In addition to this prior knowledge about the tumor lines, we also analyzed phosphoproteomic data to try to identify common signaling patterns between subsets of tumor lines. The phosphoproteomic data illustrates the heterogeneity between xenograft models of eight different patients (*7*). When we clustered proteins based on their phosphorylation ratios, we noticed that each cluster is enriched in very general biological processes (**Fig S2b**) implying that hits identified in the experimental data are not complete enough to represent disease-specific pathways.

Xenograft heterogeneity is visually obvious in **Figure S2a**, and it is clear that response to the treatments is not correlated with any single biomarker, except for the above noted TMZ-MGMT relation. For example, although *EGFR* is mutated or amplified in half of the patient set, only one tumor line is sensitive to the EGFR inhibitor (Everolimus) and EGFR gene is neither mutated nor amplified in that tumor line. These observations indicate that there may be a combinatorial effect of several biomarkers in the progression of GBM. The global and phosphoproteomic data illustrate that expression and phosphorylation profiles of proteins in different tumor lines are heterogeneous as well (see reference (*7*) and **Figure S2b**).

Prize Collecting Steiner Tree Problem. For a given, directed or undirected network $G(V, E, c(e), p(v))$ of node set V and edge set E, where a $p(v) \geq 0$ assigns a prize to each node $v \in V$ and $c(e) \geq 0$ that assigns a cost to each edge e ∈ E. The aim is to find a tree $T(V_T,E_T)$, by minimizing the objective function:

$$f(t) = \beta \sum_{v \notin V_T} p(v) + \sum_{e \in E_T} c(e)$$

where the first term is β times the sum of the node prizes not included in the tree T and the second part is the sum of the edge costs of T. Note that

$$\sum_{v \notin V_T} p(v) = -\sum_{v \in V_T} p(v) + const$$

so that minimizing $f(T)$ amounts to collecting the largest set of high prize vertices while minimizing the set of large cost edges in a trade-off tuned by β. As a starting point, we consider the message-passing algorithm for the PCST problem introduced in (*8*). The message-passing algorithm estimates overall costs shifts for conditioning each edge to belong to the optimum PCST from the solution of a set of equations, information which is sufficient to construct a global optimum. These equations are solved iteratively in a computationally efficient way. Here we present a generalization of the message passing algorithm designed to solve the PCST problem on directed networks (*i.e.* where in general c(e{i,j}) might be different from c({j,i})). In this variant, the optimization will be done on directed rooted trees, where choice of the root (which will be part of the candidate tree) is an external parameter of the algorithm.

Table S1 List of the most common proteins in the disease networks.

Protein	Function	Frequency in Disease Networks	Average Specificity Index (standard deviation)
TOLLIP	is an inhibitory adaptor protein within Toll-like receptors.	8	0.945 (0.013)
MAP2K1	Dual specificity protein kinase which acts as an essential component of the MAP kinase signal transduction pathway.	8	0.905 (0.021)
IRAK1	responsible for Interleukin-induced up-regulation of the transcription factor NF-kappa B.	8	0.904 (0.019)
CDH1	epithelial cadherin (E-cadherin) there is a correlation between the abnormal expression of E-cadherin and the growth and migration of this aggressive brain tumor subtype.	8	0.804 (0.043)
CRKL	Increased levels of Crk mRNA are frequently observed in WHO grade III and IV malignant gliomas	8	0.801 (0.034)
LCK	the non-receptor tyrosine kinase LCK is critically involved in fractionated radiation-induced expansion of the glioma-initiating cell population and decreased cellular sensitivity to anticancer treatments.	7	0.894 (0.025)
ERBB3	Multiple Erbb family receptors play role in GBM.	7	0.874 (0.037)
IKBKE	IKBKE is over-expressed in glioma and contributes to resistance of glioma cells to apoptosis via activating NF-κB.	7	0.946 (0.025)
ARIH2	E3 ubiquitin-protein ligase	7	0.924 (0.026)
UBE2L3	Ubiquitin-conjugating enzyme E2.	7	0.804 (0.014)
STAT6	Has a role in enhancing cell proliferation and invasion in GBM	6	0.997 (0.005)
BTRC	plays important roles in regulating cell cycle checkpoints, and has also emerged as an important player in protein translation, cell grow and survival.	6	0.73 (0.054)

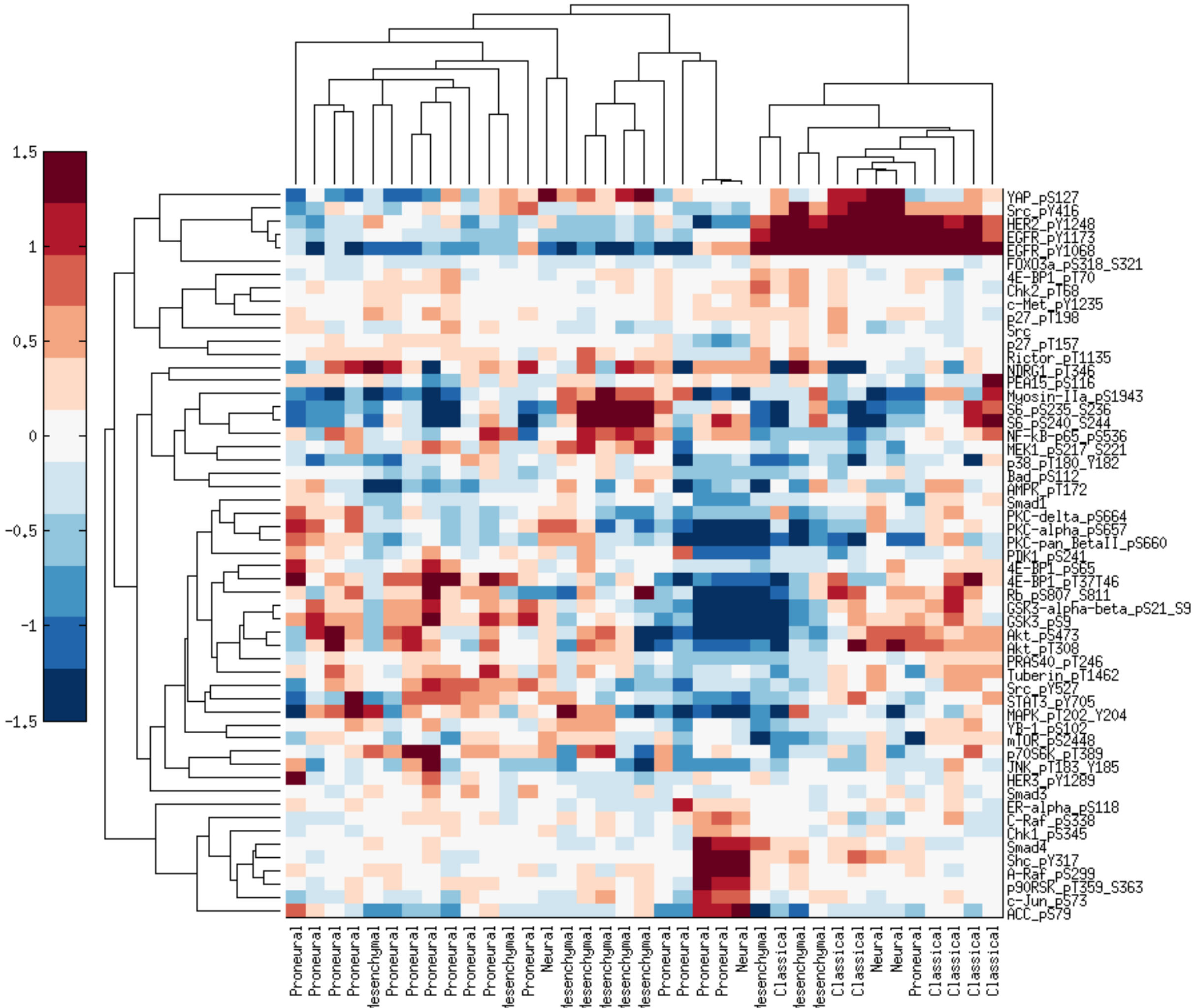

Figure S1. RPPA data of phosphorylated proteins in TCGA those have a subtype assignment from transcriptional data.

a

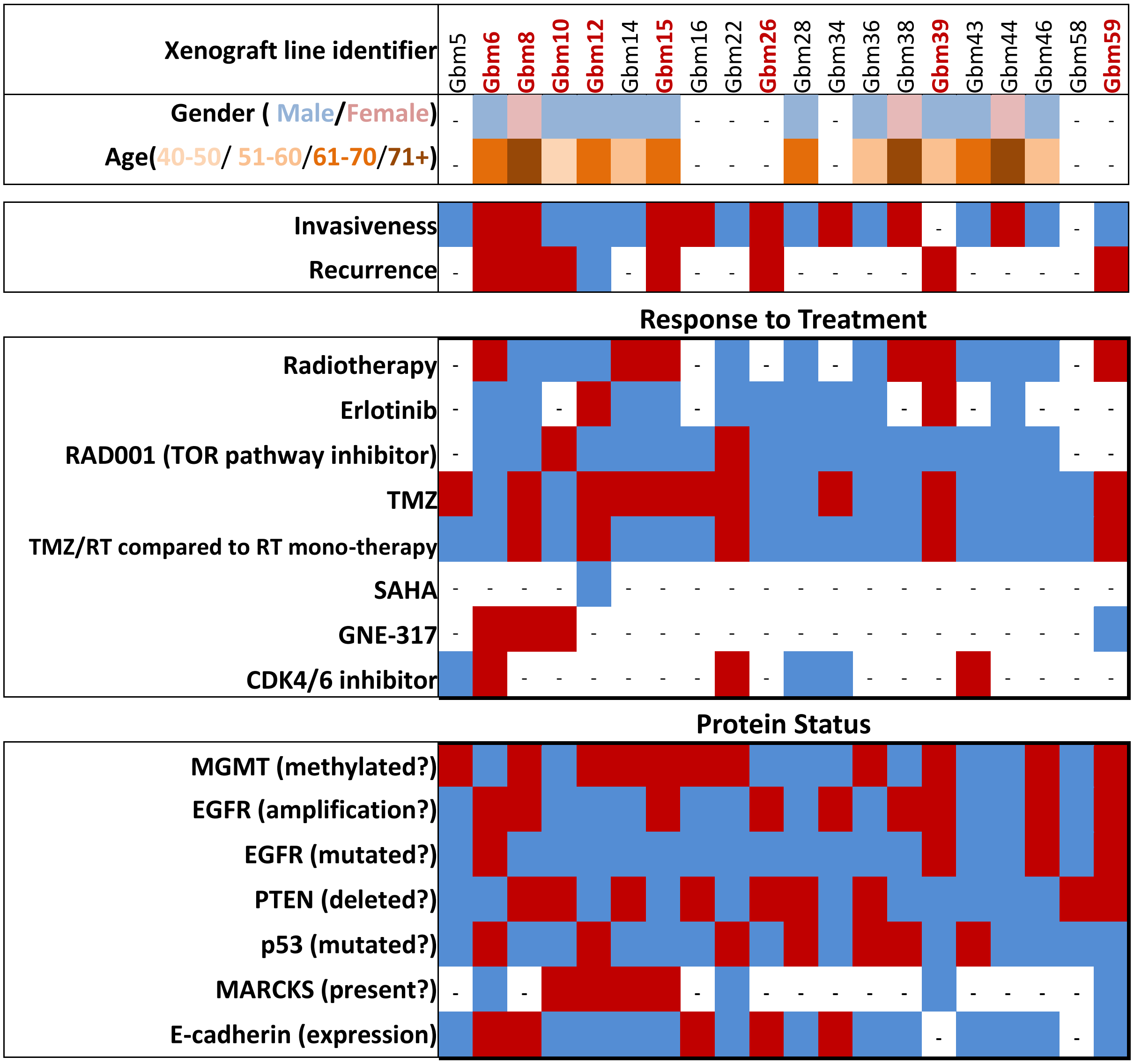
Xenograft line identifier
Gbm5
Gbm6
Gbm8
Gbm10
Gbm12
Gbm14
Gbm15
Gbm16
Gbm22
Gbm26
Gbm28
Gbm34
Gbm36
Gbm38
Gbm39
Gbm43
Gbm44
Gbm46
Gbm58
Gbm59
Gender (Male/Female)
Age(40-50/ 51-60/61-70/71+)
Invasiveness
Recurrence
Response to Treatment
Radiotherapy
Erlotinib
RAD001 (TOR pathway inhibitor)
TMZ
TMZ/RT compared to RT mono-therapy
SAHA
GNE-317
CDK4/6 inhibitor
Protein Status
MGMT (methylated?)
EGFR (amplification?)
EGFR (mutated?)
PTEN (deleted?)
p53 (mutated?)
MARCKS (present?)
E-cadherin (expression)

b

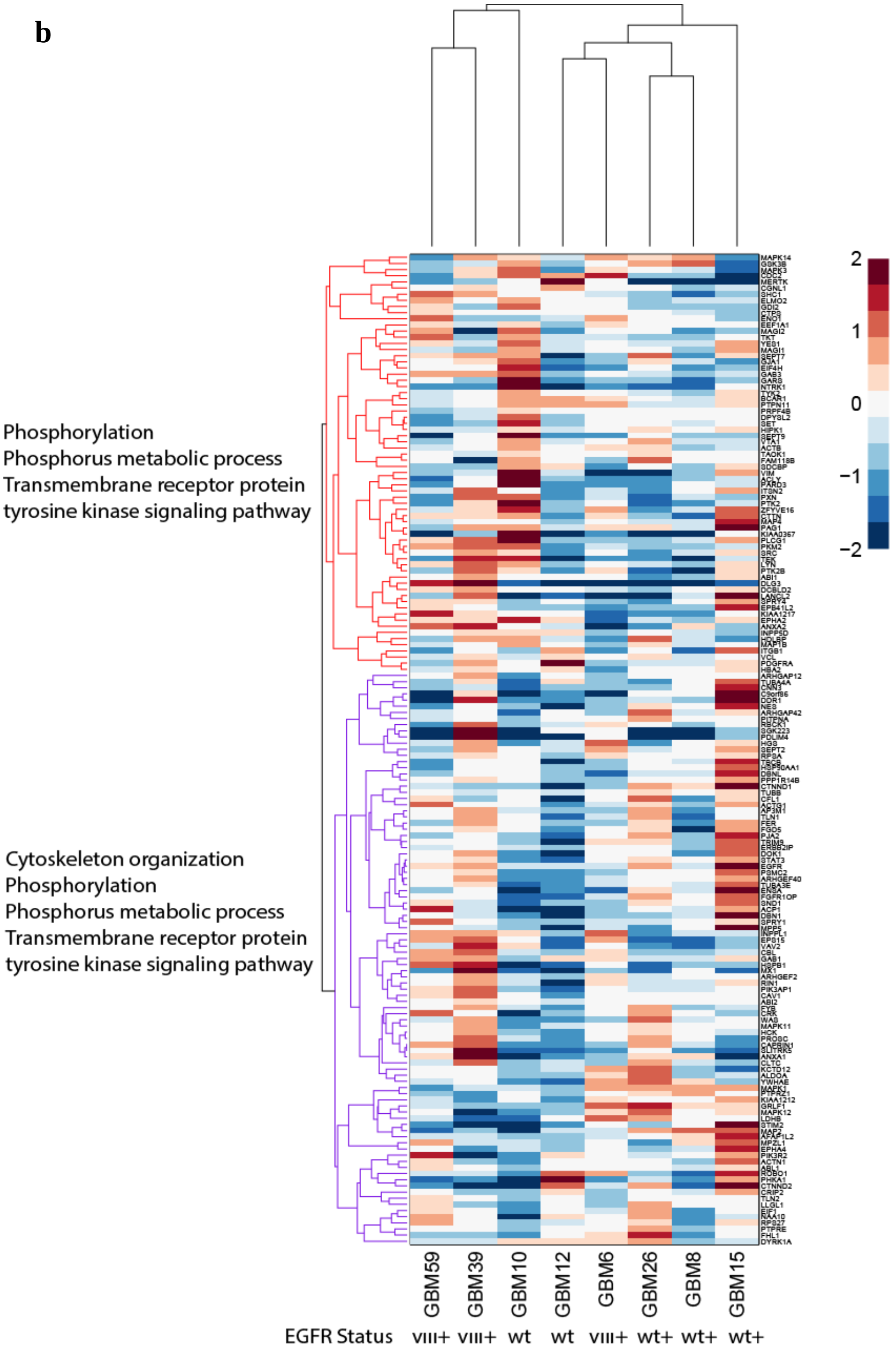

Figure S2 (a) Characteristics of the human GBM xenografts compiled from all published data for the same tumor lines. Labels of tumor lines that were analyzed in network modeling are colored dark red. The chart is divided into three parts where the first part illustrates clinical variables; such as, gender/age of patients, invasiveness (red=highly invasive, blue=moderately or non-invasive) and recurrence (red=recurrent). The second part shows the response to the treatments where red means sensitive to the respective treatment and blue means resistant. The last part gives information about the status of several biomarkers found in GBM (red=yes,

blue=no). **(b)** Heat-map of the phosphoproteomic data across 8 GBM xenograft lines. The heat-map is a representation of log2 fold changes of phosphorylation normalized to the mean of all 8 channels.

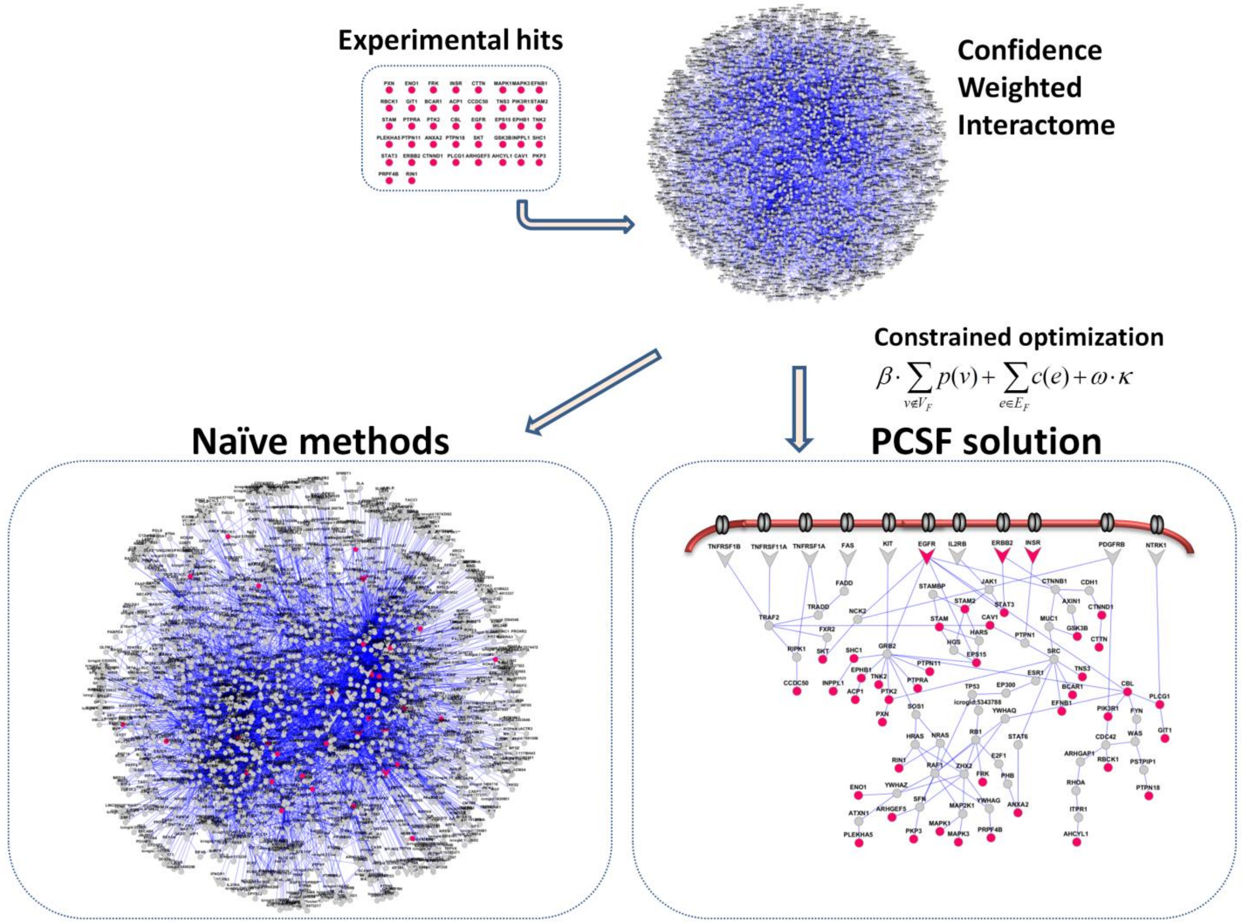

Figure S3. The conceptual flow of the prize-collecting Steiner forest algorithm. Naïve methods – here, going out to a distance of two from the terminal nodes and constraining first neighbors to be linked at least two terminal nodes – usually gives a hairball network. The PCSF algorithm provides a better way of connecting experimental hits via intermediate nodes. Additionally, it shows the signal flow starting with cell surface receptors toward downstream signaling pathways. In both solutions, red colored nodes are experimental hits and others are intermediate nodes.

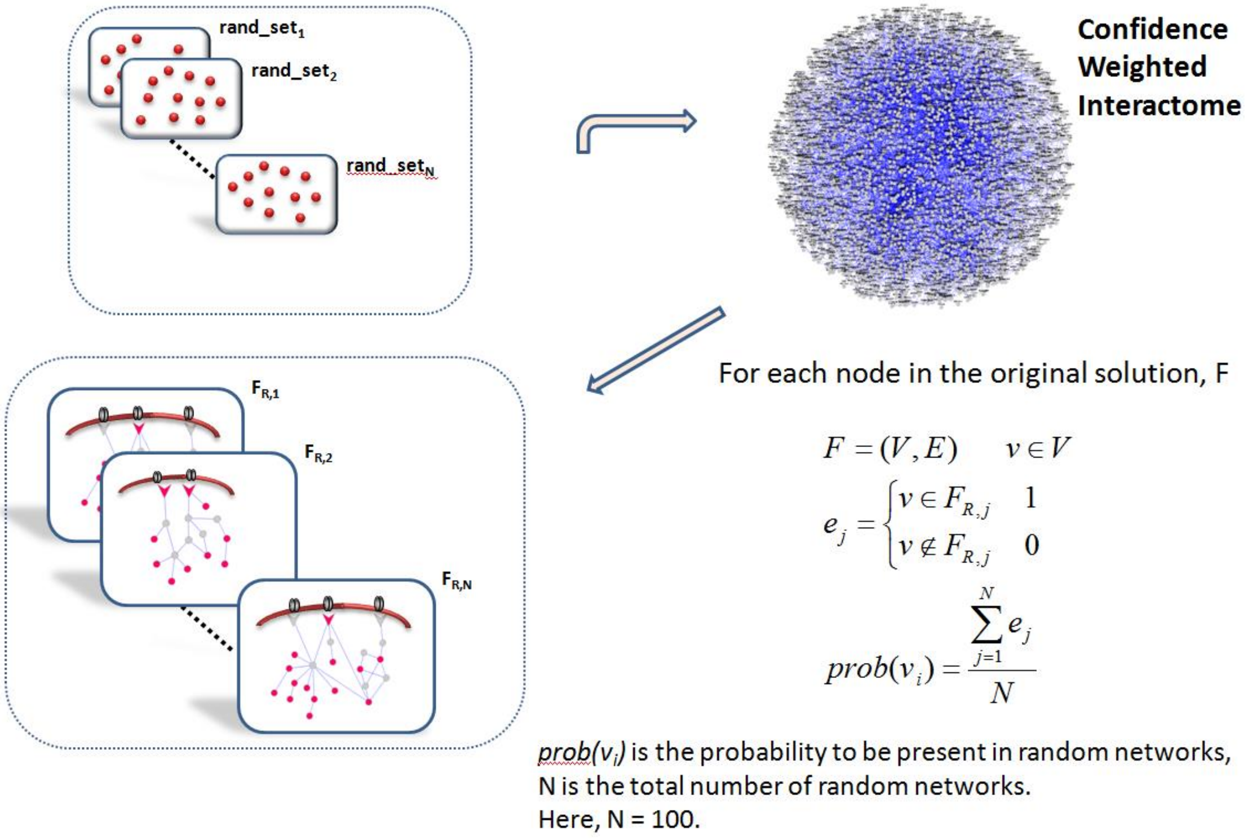

Figure S4 Randomization test. To filter the target set, 100 different terminal sets are generated for each tumor where node penalties are kept the same, but the nodes in the terminal set are selected randomly from the whole interactome. In this test, the original interactome with the original edge costs is used.

a

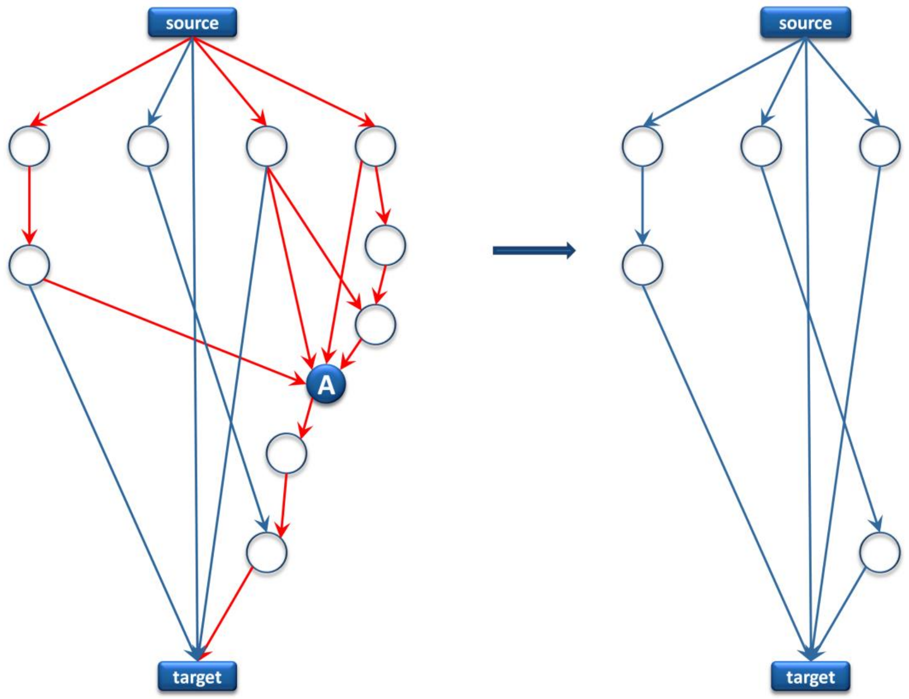

b

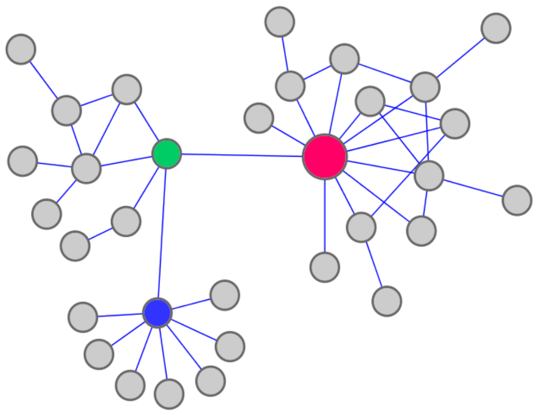

Figure S5. Methods to find high-ranking target nodes. (a) Calculation of the path damage for each node from source node to the target node. When node A is removed from the network, 5 out of 9 paths get damaged where those edges are colored in red. **(b)** Measuring the node centrality. Different centrality metrics give different ranking scores. For example in this network, although the green node does not have many neighbors, many shortest paths go through this node (low degree centrality, high betweenness node). The red node has both high betweenness and high degree centrality. The blue node has many neighbors, however it is not central in terms of betweenness (high degree centrality, low betweenness).

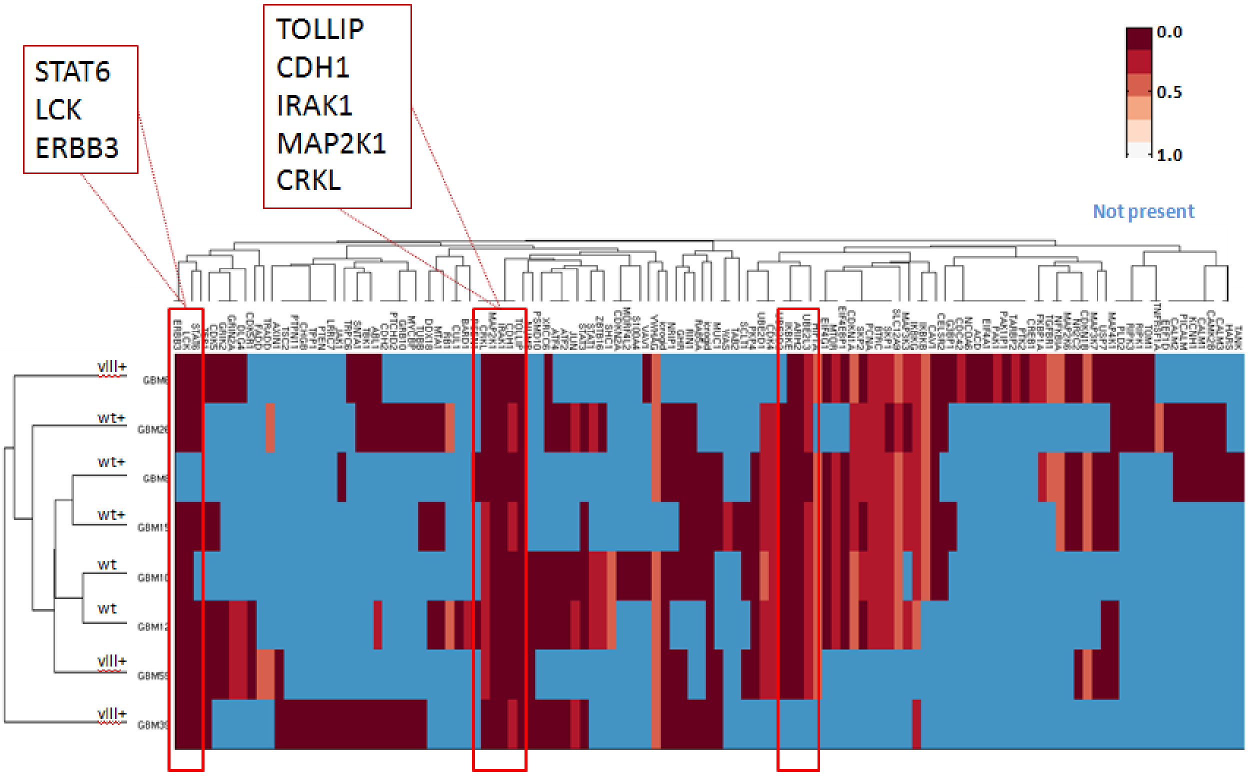

Figure S6. Top ranking proteins for each patient (union of the centrality and path damage ratio rankings). If a protein is not present in the network of a tumor line the corresponding entry is colored blue. Otherwise corresponding the entry is colored according to the p-value. The most common protein targets are highlighted in red boxes.

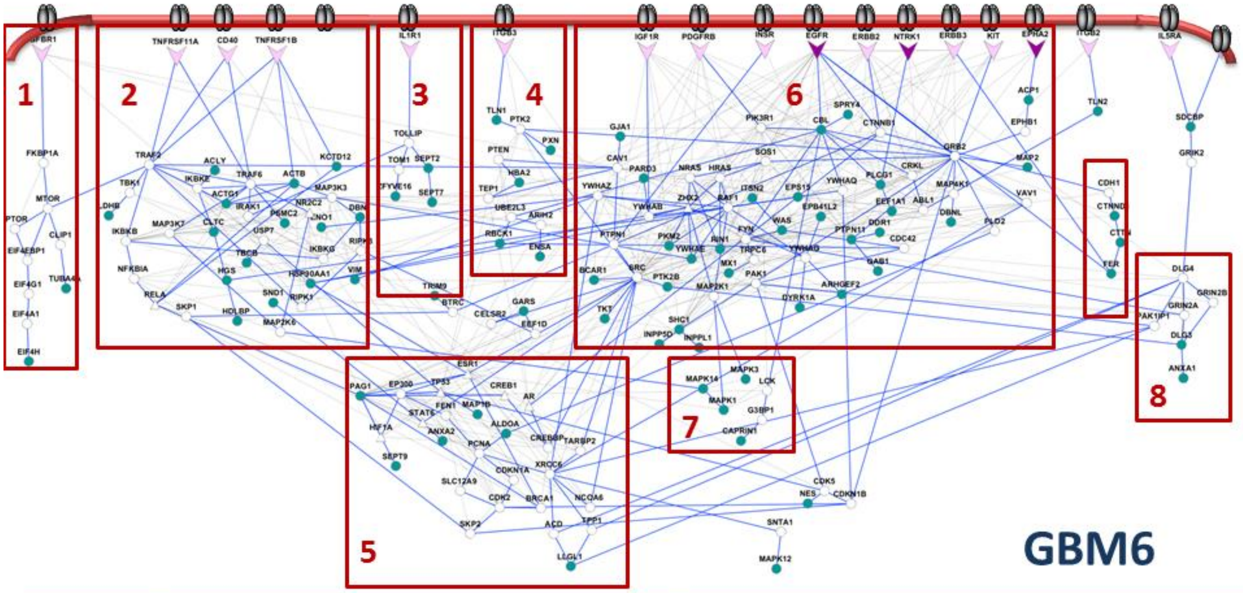

Cluster #	GO Biological Process	Cluster #	GO Biological Process
1	TOR signaling cascade<br>Regulation of protein metabolism<br>Positive regulation of cell communication	5	Regulation of DNA metabolic processes<br>Positive regulation of gene expression<br>DNA repair
2	Regulation of IKB/NFKB cascade<br>Positive regulation of NFKB transcription factor activity	6	Transmembrane protein tyrosine kinase receptor signaling pathways<br>Ras protein signal transduction<br>Regulation of MAPK activity
3	Response to interleukin-1	7	Small GTPase mediated signal transduction<br>Ras protein signal transduction
4	Endothelial cell migration<br>Angiogenesis	8	Learning<br>Glutamate signaling pathway

Figure S7 The disease network of GBM6. Nodes are clustered with the edge-betweenness clustering algorithm. Each cluster is enriched in multiple biological processes, which are tabulated in the lower panel of the figure. Nodes colored cyan are terminals, white are Steiner nodes. Arrow shaped nodes are cell surface receptors. If they were already in the terminal set, they are colored purple, if not they are colored pink.

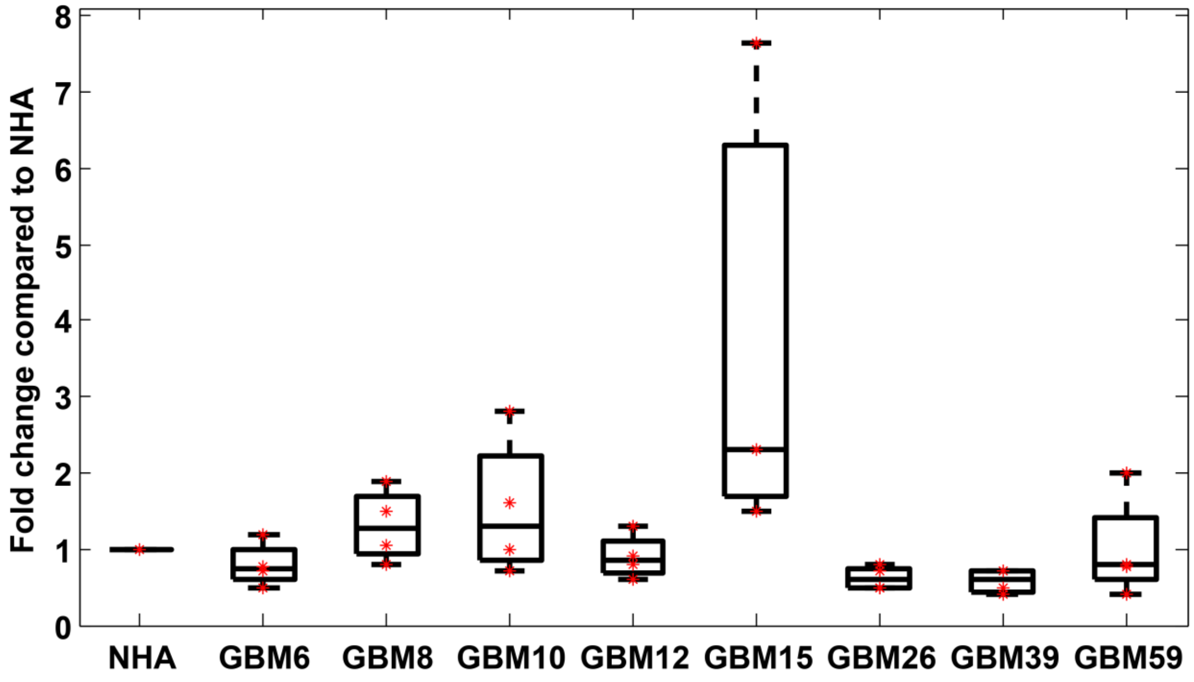

Figure S8 Boxplot of protein abundance of MEK1 calculated from Western blotting across 8 eight GBM xenograft lines (four biological replicates).

Supplementary References

1. B. L. Carlson, P. T. Grogan, A. C. Mladek, M. A. Schroeder, G. J. Kitange, P. A. Decker, C. Giannini, W. Wu, K. A. Ballman, C. D. James, J. N. Sarkaria, Radiosensitizing effects of temozolomide observed in vivo only in a subset of O6-methylguanine-DNA methyltransferase methylated glioblastoma multiforme xenografts. *International journal of radiation oncology, biology, physics* **75**, 212-219 (2009); published online EpubSep 1 (10.1016/j.ijrobp.2009.04.026).
2. L. Yang, M. J. Clarke, B. L. Carlson, A. C. Mladek, M. A. Schroeder, P. Decker, W. Wu, G. J. Kitange, P. T. Grogan, J. M. Goble, J. Uhm, E. Galanis, C. Giannini, H. A. Lane, C. D. James, J. N. Sarkaria, PTEN loss does not predict for response to RAD001 (Everolimus) in a glioblastoma orthotopic xenograft test panel. *Clinical cancer research : an official journal of the American Association for Cancer Research* **14**, 3993-4001 (2008); published online EpubJun 15 (10.1158/1078-0432.CCR-07-4152).
3. J. S. Jarboe, J. C. Anderson, C. W. Duarte, T. Mehta, S. Nowsheen, P. H. Hicks, A. C. Whitley, T. D. Rohrbach, R. O. McCubrey, S. Chiu, T. M. Burleson, J. A. Bonner, G. Y. Gillespie, E. S. Yang, C. D. Willey, MARCKS regulates growth and radiation sensitivity and is a novel prognostic factor for glioma. *Clinical cancer research : an official journal of the American Association for Cancer Research* **18**, 3030-3041 (2012); published online EpubJun 1 (10.1158/1078-0432.CCR-11-3091).
4. J. N. Sarkaria, B. L. Carlson, M. A. Schroeder, P. Grogan, P. D. Brown, C. Giannini, K. V. Ballman, G. J. Kitange, A. Guha, A. Pandita, C. D. James, Use of an orthotopic xenograft model for assessing the effect of epidermal growth factor receptor amplification on glioblastoma radiation

response. *Clinical cancer research : an official journal of the American Association for Cancer Research* **12**, 2264-2271 (2006); published online EpubApr 1 (10.1158/1078-0432.CCR-05-2510).

5. J. N. Sarkaria, L. Yang, P. T. Grogan, G. J. Kitange, B. L. Carlson, M. A. Schroeder, E. Galanis, C. Giannini, W. Wu, E. B. Dinca, C. D. James, Identification of molecular characteristics correlated with glioblastoma sensitivity to EGFR kinase inhibition through use of an intracranial xenograft test panel. *Molecular cancer therapeutics* **6**, 1167-1174 (2007); published online EpubMar (10.1158/1535-7163.MCT-06-0691).
6. L. J. Lewis-Tuffin, F. Rodriguez, C. Giannini, B. Scheithauer, B. M. Necela, J. N. Sarkaria, P. Z. Anastasiadis, Misregulated E-cadherin expression associated with an aggressive brain tumor phenotype. *PLoS One* **5**, e13665 (2010)10.1371/journal.pone.0013665).
7. H. Johnson, A. M. Del Rosario, B. D. Bryson, M. A. Schroeder, J. N. Sarkaria, F. M. White, Molecular Characterization of EGFR and EGFRvIII Signaling Networks in Human Glioblastoma Tumor Xenografts. *Mol Cell Proteomics* **11**, 1724-1740 (2012); published online EpubDec (
8. M. Bailly-Bechet, A. Braunstein, A. Pagnani, M. Weigt, R. Zecchina, Inference of sparse combinatorial-control networks from gene-expression data: a message passing approach. *BMC Bioinformatics* **11**, 355 (2010).
